# Molasses as a Food for Horses1Read before the Twelfth Annual Meeting of the New York State Veterinary Medical Society, at Brooklyn, September 9 and 10, 1902.

**Published:** 1902-12

**Authors:** George H. Berns

**Affiliations:** Brooklyn, N. Y.


### MOLASSES AS A FOOD FOR HORSES.[1]

By George H. Berns, D.V.S.,
BROOKLYN, N. Y.

In volume xxv., No. 11, of the *American Veterinary Review*, published in February, 1902, Dr. G. E. Griffin, Veterinarian, Artillery Corps, U. S. A., and stationed at Fort Sheridan, Ill., made a most interesting and instructive report upon the value of molasses as a food for horses. While serving with the 5th cavalry in Porto Rico from 1898 to 1901 he observed that the natives fed their ponies exclusively upon a very poor quality of grass mixed with molasses, and that a large quantity of molasses was added to their drinking water, and that the animals endured surprisingly the hard usage and brutal treatment they were subjected to by the native Porto Rican and Spaniard.

This caused him to begin a series of experiments with feeding molasses to army horses. He selected eight horses on January 1, 1899, and continued to experiment until June 1 the same year. The regular rations, consisting of twelve pounds of oats and fourteen pounds of hay per day for each horse, were gradually reduced, and cut grass mixed with molasses, diluted with 25 per cent. of water, was substituted. The animals the first day or two refused, or ate but sparingly of this substitution, but gradually became accustomed to it, and by the 10th of the month all the horses were eating thirty-five pounds of cut grass and fourteen pounds of molasses daily, when the regular rations of oats and hay were withdrawn.

From January 1st until January 17th these animals were doing the usual routine work of the garrison, amounting to five or six miles a day. Beginning on January 17th the regular amount of

[1] Read before the Twelfth Annual Meeting of the New York State Veterinary Medical Society, at Brooklyn, September 9 and 10, 1902.

work, consisting of twelve miles a day—that is, eight miles walk, two miles regulation trot, one mile slow gallop and one mile fast gallop—and carrying about two hundred and three pounds, was begun and continued every day except Sundays until the termination of the experiment.

During the first ten days, while the regular ration of hay and oats was being reduced, and the horses failing to eat the new food, each animal lost about twenty-seven pounds in weight; but, beginning on January 26th, they commenced to pick up, and by February 5th all of them regained their original weight, and continued to improve so that by March 1st each animal had gained about fifty pounds over the original weight.

Early in February it was observed that all the animals showed a tendency to constipation, and to correct this a little bran was given, which had to be repeated on an average of every two weeks.

Dr. Griffin also experimented with four horses which had been on the sick report for a long time suffering from chronic indigestion and its consequent loss of condition, scratches, skin abrasions, etc. On February 3d all of them were suddenly deprived of their grain and hay and put on a ration of six pounds of molasses and twenty pounds of grass daily. They refused the new food for two days, but on the sixth day they cleaned out their mangers and continued to feed well. These animals commenced to pick up at once, and within a few weeks had improved so wonderfully that their drivers failed to recognize them.

Dr. Griffin concludes his interesting article by saying that all the horses partaking of the molasses improved in spirit, coat, condition, wind, and flesh, looked better than any other horses in the garrison, and he draws the following conclusions:

1. Thirty-five pounds of grass and from thirteen to fifteen pounds of molasses as a daily allowance is sufficient to maintain a horse of one thousand pounds weight in good condition in a climate similar to that of Porto Rico.

2. Sudden change from dry food to this ration is not at all injurious and does not derange the digestive apparatus.

3. That horses on this ration appear to be able to do more work and present a much better appearance.

4. That the cost of feeding is reduced from about twenty-seven cents per day to fifteen cents per day.

Dr. Liautard, who has spent the last three or four years in Paris, France, but continues to take an active interest in veterinary matters in this country, having read Dr. Griffin's article on

"Molasses as a Food for Horses," made a thorough investigation of the subject in the French Capital and publishes his discoveries in a leading article in the *American Veterinary Review*, July, 1902, vol. xxvi., No. 4. Dr. Liautard recently visited the French horse show and noticed a little side exhibition where molasses bread and molasses biscuits were sold. He attended a meeting of the Société Centrale where the question of molasses feeding became the subject of a most interesting discussion, in which very important facts were presented by one, Mr. Lavalard, who is director of the cavalry of the Compagnie Generale des Omnibus, and having charge of fifteen hundred horses.

Mr. Lavalard has had all his horses, which weigh between 500 and 600 kilog. each, fed on the following rations for a long time: Crushed oats, corn, and beans mixed, 7 kilog.; molassed peat, 2 kilog.; cut straw between 3 and 4 kilog., with no hay whatever. He reports most gratifying results, and said that the cost of feeding had been reduced from about fifty-five cents to a fraction over thirty-five cents.

Dr. Liautard says that many compounds of molasses have been made, but that molassed peat, sold under the name of molassine, is to-day extensively used not only in Paris, but in the German, Austrian, and Russian armies, and from the general total of observations made the following conclusions have been adopted:

1. That there is no danger or inconvenience whatever to give in the daily rations of a horse at least 6 kilog. of molassed peat of good quality in the proportion of 20 per cent. of peat and 80 per cent. of molasses.

2. That to the extent of 1 kilog. at least, molassed peat takes absolutely the place of the same quantity of good oats.

3. That by this change of diet the general condition, muscular power, energy to work, and health not only remain perfect, but that the coat looks better and more glossy.

As veterinarian to the firm of Arbuckle Bros., sugar refiners and coffee dealers, of this city, employing about one hundred head of heavy draught horses, which have been fed to a very large extent upon molasses for over one year, I have had an excellent opportunity to observe its effects, not only as to its nutritive value as a food, but also as to its action upon the general health of horses.

Arbuckle Bros. having shipped cargoes of molasses to Europe to be used as food for horses, decided to experiment with it in their own stables, and on April 15, 1901, selected two extremely unthrifty-looking animals as subjects.

No. 1 weighed 900 pounds; No. 2 weighed 940 pounds. Both animals were photographed, and their pictures plainly show their miserable condition.

Without any preparation whatever they were suddenly deprived of their usual rations of hay and oats, and received one quart of molasses diluted with three quarts of water and mixed with five pounds of cut hay three times a day, and were allowed to drink all the water they wanted, but received no long hay nor any other kind of food, and no exercise. For a day only they minced over their rations; the second day they cleaned out their mangers, and continued to eat well until the termination of the experiment, which lasted six weeks. It was noticed that during this time both animals consumed larger quantities of water, but showed no symptoms of constipation or diarrhœa, or any other signs of digestive disturbances; their general condition improved wonderfully in two weeks' time. No. 1 had gained 40 pounds in weight; No. 2 had gained 45 pounds in weight; in four weeks' time No. 1 had gained 95 pounds in weight and No. 2 had gained 102 pounds in weight. Both animals were shedding their old coats, and by June 1st No. 1 weighed 1075 pounds and No. 2 weighed 1086 pounds, and both showed beautiful new glossy coats, and when led from the stable were full of play, life, and energy, and could not have been recognized as the same animals. One was sold to a retail grocer in this city, and the other was retained by the firm and sent to work. Both animals remained in good condition and did their work well, in spite of the fact that they had not been exercised in six weeks.

The success of these experiments caused the firm to begin the systematic feeding of all their heavy truck horses, one hundred in number, with molasses on June 1, 1901. At this time all of the horses were in fair working condition; average weight of each about seventeen hundred pounds, and doing an average day's work of ten hours, seldom going any faster than a walk, but pulling very heavy loads—two-horse trucks carrying twenty-five barrels of sugar, or about five and one-half tons; three-horse trucks carrying thirty-five barrels of sugar, or about seven and three-quarter tons. Each animal received a mixture of one quart of molasses diluted with three quarts of water and thoroughly mixed with six pounds of good quality of cut hay, one and one-half quarts of corn meal, and two and one-half quarts of coarse bran for breakfast, five quarts of dry oats in the middle of the day while in harness, and the same mixture and quantity of molasses, cut hay, corn, and bran for supper, with eleven pounds of long hay added for the night.

Under these rations, and doing the same amount of work, all the animals gradually improved in condition and gained in weight, their coats became slick and glossy, and to-day there are no finer-looking truck-horses in Greater New York. Their general health has been excellent, and cases of acute indigestion or spasmodic colic have been very rare in Arbuckles' stables during the last fourteen months, where they were quite frequent in former years.

The success of Arbuckles', together with the report published by Drs. Liautard and Griffin, prompted me to try molasses feeding among my own horses and some of the patients in the infirmary.

Two patients, one a brown gelding greatly reduced in condition from the acute pain of an open joint, and the other a bay truck-horse, poor in flesh and very lame from painful ringbone on a hind extremity, together with my own five driving-horses, were selected for the experiment.

On August 1st, patient No. 1, brown gelding, weighed 1250 pounds; patient No. 2, bay truck-horse, weighed 1140 pounds. They received three pounds of molasses diluted with three quarts of water and mixed with six pounds of cut hay, and two and one-half pounds of corn meal, and two and one-half pounds coarse bran, morning and evening, and about three pounds of long hay at night. My five driving-horses received the same rations, with the addition of four quarts of dry oats in the middle of the day.

The two patients improved very rapidly in condition, and on August 15th, No. 1, brown gelding, weighed thirteen hundred and twenty pounds, having gained seventy pounds. No. 2 weighed twelve hundred pounds, showing a gain of sixty pounds. September 1st, No. 1, brown gelding, weighed fourteen hundred and fifty pounds, and No. 2, bay truck-horse, weighed twelve hundred and fifty pounds; total gain in thirty-one days, No. 1, brown gelding, two hundred pounds; No. 2, bay truck-horse, one hundred and ten pounds.

I very much regret that my driving-horses were not weighed when the change of diet was commenced, but they have done their usual amount of work, they drive up well, do not fag out in travelling long distances, and are certainly looking much better than they were five or six weeks ago when they were getting from twelve to fifteen quarts of white oats and about fifteen pounds of long hay daily, and, as in all other experiments, not the slightest signs of indigestion were noticed.

From the above described practical feeding experiments conducted in different countries and under widely different conditions it is safe to conclude:

1. That molasses of a good quality is a most nutritious food for horses, easily digested and assimilated, and will, in many cases, correct faulty digestive processes.

2. That one quart of molasses at a cost of three cents will take the place of from three to four quarts of good quality oats at a cost of from four and one-half to six cents.

3. That a sudden change from dry oats to molasses mixed with other food-stuffs is perfectly safe and causes no disturbances of the digestive organs.

4. That molasses-fed horses will do fully as much work, and at the same time remain, as a rule, in much better general condition than animals fed on dry food, while the cost of feeding is reduced from 25 per cent. to 33 per cent.

At first glance it seems very strange that an article of a semi-solid consistency, sweet of taste, dark in color, sticky and unpleasant to handle, should be able to take the place of nice, clean, fine-looking oats, which, from time immemorial, has been the recognized standard food for horses; but when we study the subject for a few moments from a physiological point of view, and at the same time consider the conditions under which our work-horses are kept, most all of you will probably agree with me that it is not at all surprising, but perfectly logical.

It is an established physiological fact that all foods entering the animal body by way of the mouth must be reduced to a soluble condition before absorption into the general circulation can possibly take place.

It is also an undisputed fact that starches must be converted into sugar or glucose before absorption or assimilation takes place.

Oats being a hard, dry substance, and largely composed of starch, require a great deal of preparation on the part of the animal before they are reduced to a soluble condition and the starch converted into sugar. They must be thoroughly masticated or triturated by the molar teeth and mixed with the various salivary secretions before they are fit for stomach digestion.

In the stomach and small intestines the gastric, pancreatic, and intestinal juices, together with the bile from the liver, must be brought into contact with it, each in its turn and each doing its allotted work, before the wonderful chemical transformation, reducing the hard, dry oats into the nutritious pabulum called chyme, is accomplished.

The question now arises: Is this intricate process always accomplished in the horse? Anyone who has had experience with hard-working draught-horses will agree with me that quantities of whole oats, or partly crushed oats in various stages of decomposition and putrefaction, are found in the manure of many horses, and in bad cases the manure is of an unnatural color, very offensive, and of a semisolid consistency. This proves that the process of digesting oats is imperfectly performed, that they are not always reduced to a soluble assimilable condition, and, therefore, not only lose much of their nutritive value as a food, but remain in the intestinal tract, decomposing, fermenting, generating gases, and causing colics and more frequent milder forms of intestinal disturbances.

Many such horses are always hungry and will eat unlimited quantities of oats and receive but little benefit therefrom, and remain in poor condition, while others become slowly poisoned from the undigested fermenting residue constantly present in the alimentary canal, and have but little appetite for food of any description.

Now, let us consider the conditions under which our work-horses are kept: many of them work from eight to twelve hours a day, draw heavy loads, and are obliged to travel many miles; at five or six in the morning they receive a full meal of dry oats, and after feeding are allowed all the water they desire to drink, and are immediately placed in harness and kept steadily at work until 11 or 12 o'clock, when they receive their noon meal, consisting of from four to eight quarts of dry oats fed out of a canvas nose-bag; as soon as the meal is finished they must again assume their regular drudgery, and continue until 5, 6, or 7 o'clock at night, returning to their stables completely tired out and greatly exhausted. From four to eight quarts of oats and from sixteen to twenty pounds of hay are placed in front of each horse, and he is left for the night.

The poor animal is hungry when his noonday meal is served, and he is hungry and exhausted when his evening rations are placed in front of him. His system demands nutrition, and he greedily devours his oats and hay, but unfortunately does not take time to masticate it properly, and the above-described digestive disturbances, resulting in malnutrition and malassimilation, are the inevitable results if he is constantly fed on dry food, which is difficult to digest, and, as we have seen, requires so much preparation.

Molasses, on the other hand, while it contains a larger quantity of nutritive material than oats, is in a soluble condition the moment it enters the mouth, and, therefore, readily digested and absorbed. If properly diluted with water and thoroughly mixed with a liberal quantity of cut hay, a little corn meal and bran, it serves to soften the other foods and renders their digestion much easier, and I believe that most all work-horses, at least, would be greatly benefited if they received from two to three quarts of molasses a day.

